# Atrial Fibrillation and Heart Failure

**DOI:** 10.3390/life14050572

**Published:** 2024-04-29

**Authors:** Gabriel Cismaru, Alina-Gabriela Negru

**Affiliations:** 1 5th Department of Internal Medicine, Cardiology Rehabilitation, Iuliu Hatieganu University of Medicine and Pharmacy, 400012 Cluj-Napoca, Romania; 2 Department of Cardiology, University of Medicine and Pharmacy, 300041 Timișoara, Romania

Recent years have witnessed progress in the management of heart failure through the development of SGLT2 inhibitors and angiotensin receptor neprilysin inhibitors. Additionally, advancements in catheter ablation techniques have improved the treatment of atrial fibrillation. The two diseases are interconnected. Atrial fibrillation may result in heart failure and arrhythmia-induced cardiomyopathy, while heart failure frequently complicates atrial arrhythmias such as atrial fibrillation or ventricular arrhythmias [1].

Heart failure patients pose a particular challenge when it comes to using pharmacological treatment for rhythm management of atrial fibrillation. Due to their detrimental impact on cardiac contractility, class IC antiarrhythmic drugs such as flecainide and propafenone are not recommended. Amiodarone is authorized for the treatment of heart failure with reduced ejection fraction (HFrEF); however, it is associated with a wide range of adverse effects if used for a long period of time. Catheter ablation may be required if patients with atrial fibrillation fail to respond to drug therapy or develop adverse reactions. Pulmonary vein isolation is regarded as the essential component of catheter ablation for atrial fibrillation in conjunction with other methods for substrate treatment in persistent forms of AF [1].

Nevertheless, there are still areas of knowledge that need to be filled regarding the proper treatment of people who have both atrial fibrillation (AF) and heart failure (HF): What is the most effective approach to measuring the extent of atrial fibrosis in patients diagnosed with heart failure? What is the most effective ablation approach for substrate modification in persistent atrial fibrillation? What is the effect of left atrial appendage occlusion in patients with atrial fibrillation (AF) and heart failure (HF)? Is the combination of CRT implantation with AV node ablation more effective than pulmonary vein isolation in patients with reduced left ventricular ejection fraction and heart failure?

The initial manuscript of the Special Issue authored by Anne-Kathrin Henckell et al. describes an atrial rhythm caused by increased vagal tone, defined by a negative P wave in the inferior leads. Low atrial rhythm was identified in 0.6% of the 24,316 children included in the study. It could be linked to an atrial septal defect and the persistence of a left superior vena cava, both of which are associated with a higher likelihood of developing atrial fibrillation in adulthood [2]. 

Maria Iovanescu and coworkers conducted a study on the geometry and function of the left atrium and other heart chambers using modern echocardiography techniques. The study included 41 patients with atrial fibrillation and 47 patients with sinus rhythm. Every patient exhibited dilated cardiomyopathy. Indexed volumes in the left atrium (LA) were greater in patients with atrial fibrillation compared to patients with sinus rhythm. Additionally, patients with atrial fibrillation had a considerably lower LA emptying fraction and reduced LA global longitudinal strain during the reservoir phase [3]. 

The next three publications focus on different approaches to treating atrial fibrillation, namely the use of medications, cardioversion, and catheter ablation. Pedro Garcia Bras and his colleagues conducted an analysis of Sacubitril/Valsartan treatment in patients with heart failure and a reduced ejection fraction. Their study conducted on 42 patients demonstrated that Sacubitril/Valsartan resulted in significant improvements in LA phasic strain and strain rates, with a 17.5% enhancement in LA conduit strain and a 40% enhancement in LA reservoir strain. In addition, there was a 51.4% improvement in LA contraction strain and a 31.7% improvement in LA contraction strain rate [4].

Josip Kedzo et al. conducted a study on 41 patients utilizing brain MRI to investigate the relationship between persistent atrial fibrillation (AF), brain perfusion, and cognition before and after cardioversion. Baseline brain perfusion measures did not differ between patients with AF and control participants. Nevertheless, the procedure of restoring and maintaining the sinus rhythm was linked to an improvement in MRI brain perfusion parameters in all examined regions. This correlation was further validated by noting the absence of improvement in cerebral perfusion in patients who did not successfully restore sinus rhythm after electrical cardioversion [5].

Min Suk Choi et al. conducted a thoracoscopic ablation procedure on 20 patients with persistent atrial fibrillation and then assessed the results. Two to 3.5 ablation lines were used to create blockage of conduction to the pulmonary vein. This approach is considered the safest and most successful method. The authors conducted a total of six ablation lines around each pulmonary vein antrum, even after confirming the conduction block. However, an increased number of ablation lines may result in a higher likelihood of complications, such as pulmonary vein stenosis. Surgeons should reduce the number of unnecessary ablation lines [6].

Hence, we should regard this Special Issue as an important step towards the future management of the association between heart failure and atrial fibrillation. Readers will definitely find this Special Issue beneficial, and we welcome new ideas to improve the current version.

## References

[1] Bergau L., Bengel P., Sciacca V., Fink T., Sohns C., Sommer P. (2022). Atrial Fibrillation and Heart Failure. J. Clin. Med..

[2] Henckell A.-K., Gusetu G., Rosu R., Ciobanu D.M., Istratoaie S., Muresan L., Lazea C., Pop D., Cismaru G., Bârsu C. (2022). Low Atrial Rhythm in a Large Cohort of Children from Transylvania, Romania. Life.

[3] Iovănescu M.L., Hădăreanu D.R., Toader D.M., Florescu C., Istrătoaie O., Donoiu I., Militaru C. (2023). The Impact of Atrial Fibrillation on All Heart Chambers Remodeling and Function in Patients with Dilated Cardiomyopathy—A Two- and Three-Dimensional Echocardiography Study. Life.

[4] Brás P.G., Gonçalves A.V., Branco L.M., Moreira R.I., Pereira-Da-Silva T., Galrinho A., Timóteo A.T., Rio P., Leal A., Gameiro F. (2023). Sacubitril/Valsartan Improves Left Atrial and Ventricular Strain and Strain Rate in Patients with Heart Failure with Reduced Ejection Fraction. Life.

[5] Kedžo J., Kojundžić S.L., Guić M.M., Tandara L., Brešković T., Jurišić Z. (2023). Association of Electrical Cardioversion with Brain Perfusion and Cognitive Function in Patients with Atrial Fibrillation. Life.

[6] Choi M.S., Lee Y., Jeong D.S. (2023). The Minimum Number of Ablation Lines for Complete Isolation of the Pulmonary Veins during Thoracoscopic Ablation for Atrial Fibrillation. Life.

